# An Integrated Tiered Service Delivery Model (ITSDM) Based on Local CD4 Testing Demands Can Improve Turn-Around Times and Save Costs whilst Ensuring Accessible and Scalable CD4 Services across a National Programme

**DOI:** 10.1371/journal.pone.0114727

**Published:** 2014-12-09

**Authors:** Deborah K. Glencross, Lindi M. Coetzee, Naseem Cassim

**Affiliations:** 1 Department of Molecular Medicine and Haematology, School of Pathology, Faculty of Health Sciences, University of the Witwatersrand, Johannesburg, South Africa; 2 Charlotte Maxeke Johannesburg Academic Hospital, National Health Laboratory Service (NHLS), Johannesburg, South Africa; Johns Hopkins Bloomberg School of Public Health, United States of America

## Abstract

**Background:**

The South African National Health Laboratory Service (NHLS) responded to HIV treatment initiatives with two-tiered CD4 laboratory services in 2004. Increasing programmatic burden, as more patients access anti-retroviral therapy (ART), has demanded extending CD4 services to meet increasing clinical needs. The aim of this study was to review existing services and develop a service-model that integrated laboratory-based and point-of-care testing (POCT), to extend national coverage, improve local turn-around/(TAT) and contain programmatic costs.

**Methods:**

NHLS Corporate Data Warehouse CD4 data, from 60–70 laboratories and 4756 referring health facilities was reviewed for referral laboratory workload, respective referring facility volumes and related TAT, from 2009–2012.

**Results:**

An integrated tiered service delivery model (ITSDM) is proposed. Tier-1/POCT delivers CD4 testing at single health-clinics providing ART in hard-to-reach areas (<5 samples/day). Laboratory-based testing is extended with Tier-2/POC-Hubs (processing ≤30–40 CD4 samples/day), consolidating POCT across 8–10 health-clinics with other HIV-related testing and Tier-3/‘community’ laboratories, serving ≤40 health-clinics, processing ≤150 samples/day. Existing Tier-4/‘regional’ laboratories serve ≤100 facilities and process <350 samples/day; Tier-5 are high-volume ‘metro’/centralized laboratories (>350–1500 tests/day, serving ≥200 health-clinics). Tier-6 provides national support for standardisation, harmonization and quality across the organization.

**Conclusion:**

The ITSDM offers improved local TAT by extending CD4 services into rural/remote areas with new Tier-3 or Tier-2/POC-Hub services installed in existing community laboratories, most with developed infrastructure. The advantage of lower laboratory CD4 costs and use of existing infrastructure enables subsidization of delivery of more expensive POC services, into hard-to-reach districts without reasonable access to a local CD4 laboratory. Full ITSDM implementation across 5 service tiers (as opposed to widespread implementation of POC testing to extend service) can facilitate sustainable ‘full service coverage’ across South Africa, and save>than R125 million in HIV/AIDS programmatic costs. ITSDM hierarchical parental-support also assures laboratory/POC management, equipment maintenance, quality control and on-going training between tiers.

## Background

In 2013 there were ∼5.6 million South Africans infected with HIV [1]. Following the implementation of the South African ‘Comprehensive Care, Management and Treatment (CCMT) of HIV and AIDS’ [2] in 2003, at least 1.2 million eligible HIV positive people had been enrolled to receive Antiretroviral Therapy (ART) by 2007 [3]. Further initiatives to improve access to treatment were revealed in the National Department of Health (NDOH) ‘HIV & AIDS and STI Strategic Plan for South Africa 2007–2011 (NSP) [4], [5]. This was closely followed by the ‘HIV Counselling and Testing (HCT) Campaign’, which aimed to test a further 15 million individuals by July 2011 and facilitated 2 million eligible HIV+ people for enrolment onto ART by December 2012 [1], [6]. A 20-year strategy to reverse the burden of HIV, Sexually Transmitted Infections and Tuberculosis, in South Africa has also been published [6], [7], including plans for the introduction of National Health Insurance (NHI) pilot districts [8].

Laboratory monitoring is required for patients enrolled onto ART initiatives. Scaling-up of ART programmes demands similar major scaling-up of laboratory capacity to meet the anticipated increased clinical service needs. The South African National Health Laboratory Service (NHLS) initially responded by providing a growing number of related service laboratories, including general pathology testing services, HIV viral load or infant diagnostics, and CD4 services [9], [10]. As increasing numbers of eligible patients present for HCT and ART however [6], additional capacity and further streamlining of current laboratory services is required. For CD4 testing, the NHLS currently provides a two-tiered, laboratory-based CD4 national service [9], structured according to volumes of tests referred to testing laboratories. Preliminary CD4 testing using laboratory equipment on mobile units was shown to be cumbersome and too expensive for routine day-to-day monitoring [11]. Point-of-care servicing, advocated and used by some groups in South Africa [12], is not included in the current NHLS repertoire of testing, but recommendations appear in a 2013 position paper entitled ‘National Strategic Plan for POC’ [13].

The aim of this study was to review current CD4 services and develop a service-model that integrates laboratory-based and POC testing to improve local turn-around time (TAT) but contain costs in South Africa. This work builds on recent congress presentations [14], [15] and previously described CD4 tiered services [9] and takes into account local NDOH primary health care initiatives [4], [16] and an earlier World Health Organisation call for ‘the development of context-specific service delivery models [17] with decentralized, flexible service delivery systems to enable reaching underserved and difficult-to-reach remote areas and ensure outreach services. Detailed analysis of related costs of the service-model proposed is published elsewhere in this journal [18].

## Materials and Methods

### Service volumes review

**Figure 1:**
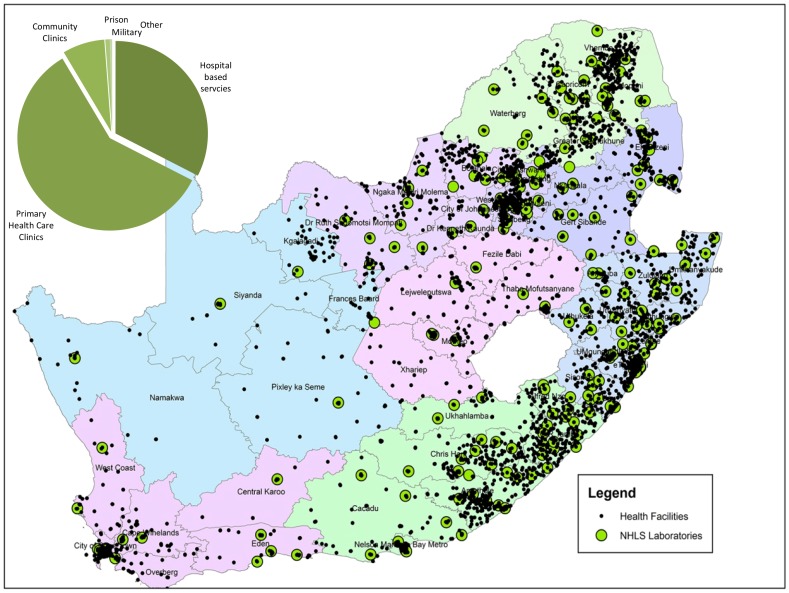
Geographical location of health and laboratory facilities in South Africa. Map to reveal geographic location of ∼4756 health facilities (as at 2011/2012); including primary care, community centers and hospital-based clinics (black dots) and 260 NHLS routine pathology service laboratories, across nine provinces and the related 52 districts. Insert reveals the proportions of different category of health facilities requesting CD4 testing (also see Table 1).

**Figure 2:**
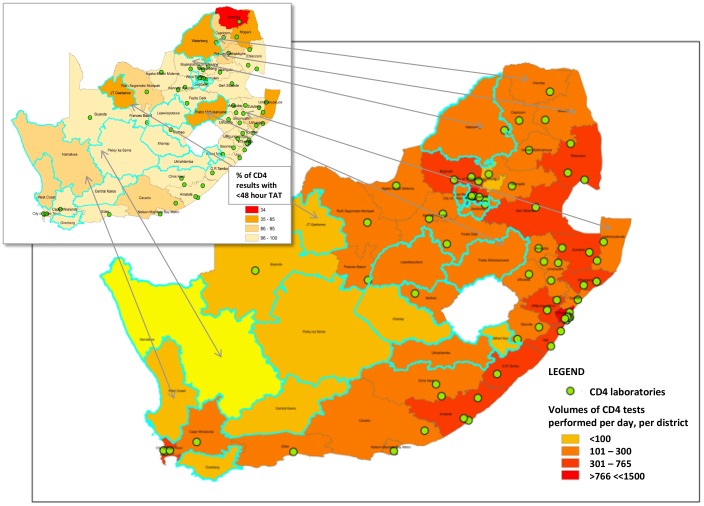
Colour-graded map indicating CD4 test volumes and laboratory-to-result turn-around-time (LTR TAT) in South Africa. Map to reveal the daily CD4 test service volumes (workload), across 52 districts in South Africa, colour-graded according to volumes of tests requested, averaged over three year from 2009–2012. Higher testing volumes (as red or orange) as well as ‘hard to reach areas’ with low testing needs (yellow, more likely to require POC testing) are revealed. Approximately 3.8 million CD4 samples were referred during 2012 to an annual average of ∼60 designated NHLS CD4 facilities (existing shown as green dots). Insert reveals proportions of reports issued within a TAT of 48-hours, across all districts, averaged over years 2009–2012. The legend here highlights districts (as red) with less than 34% of reports or 35–80% of reports (mustard orange) issued within a 48-hour TAT (see legend on figure).

**Figure 3:**
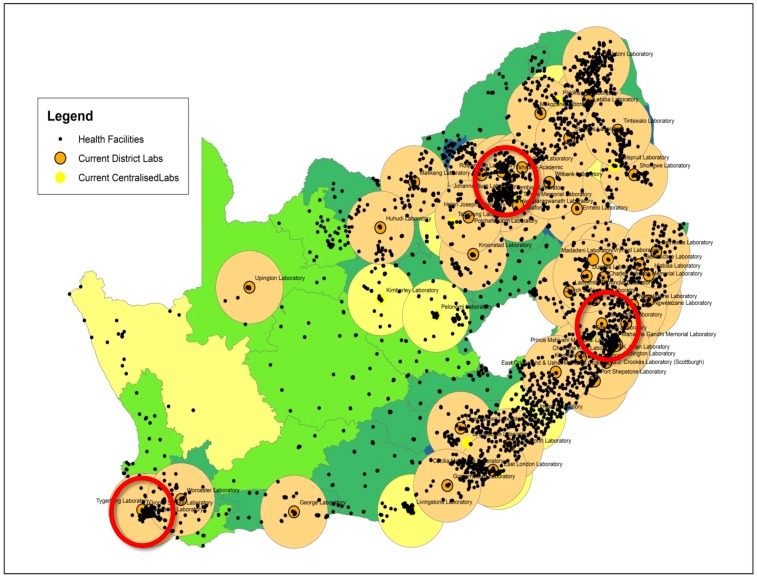
Current CD4 service coverage precincts. Map to reveal current estimated service precincts based on an averaged 100 km Euclidian radius. Areas without drawn service precincts largely coincide with districts with poorer LTR-TAT (see insert Fig. 2). Note many health care facilities that fall outside of service precincts that would benefit from implementation of additional Tier-1, 2 and 3 services. Red circles highlight relatively over-subscribed areas with multiple ‘centralised’/metro laboratories in densely populated areas. In such metropolitan areas with high testing demands, amalgamation of services and the formation of a ‘super-laboratory’ could create critical mass, consolidate on technical skills and quality control provided that transport and IT logistics are absolutely optimized.

**Figure 4:**
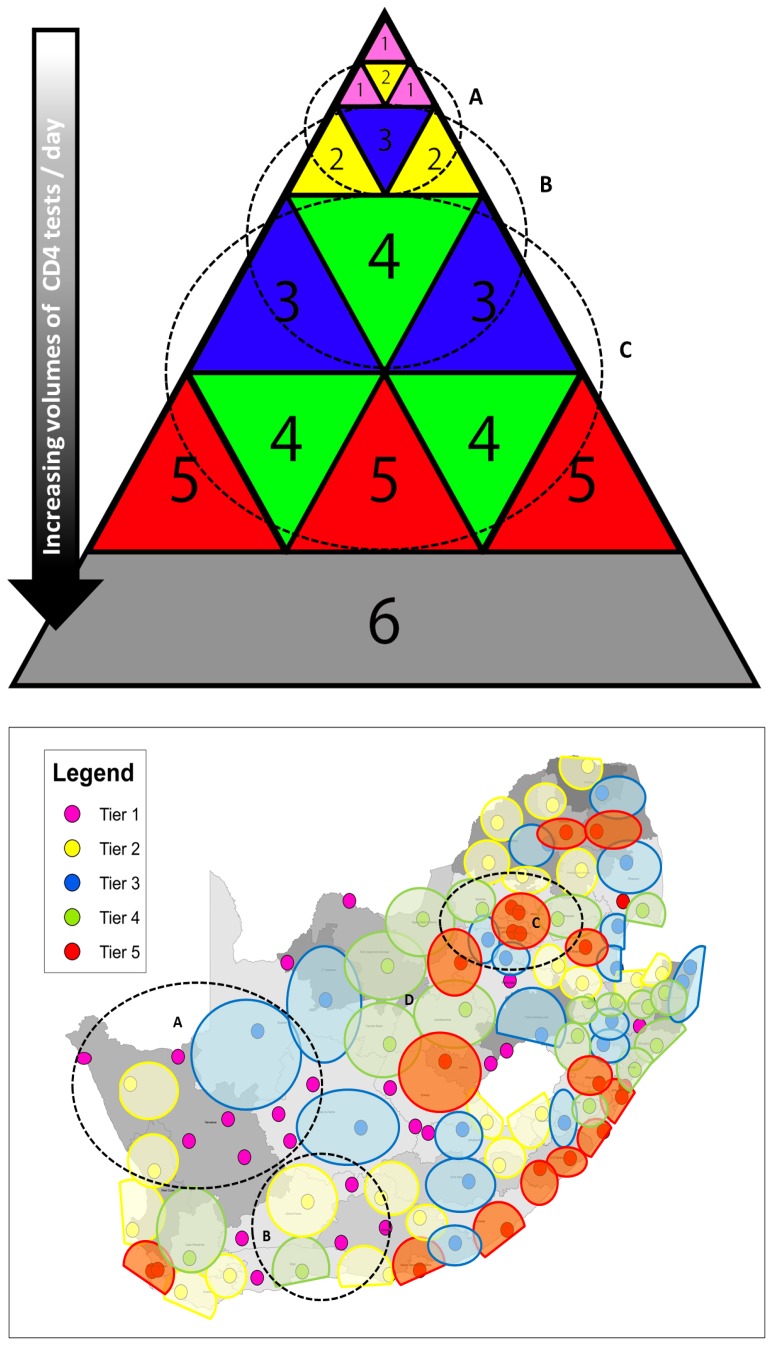
Six-tiered CD4 service framework and ideal proposed service coverage. **4a** Graphical representation of an integrated, hierarchical ‘parent’, six-tiered CD4 service approach to secure scalable, ‘full-coverage’ across a national programme. From top to base, each band represents an increasing service load from an increasing base of referring health clinics. The proposed hierarchical ‘parent’ spatial support relationship between, and within, service tiers illustrates how higher service tiers can support and interact with lower service tiers, not only in a direct hierarchical fashion, but also how geographical location of different tiers in any given region can enable ‘parent/support’ relationships. **4b** Reveals existing and ideal proposed service coverage precincts of 5 tiers of service in South Africa, based on an averaged 50–100 km radius ‘coverage-precincts’. In both 4a and 4b, ‘A’ and ‘B’ reveal examples of the envisaged integrated support relationships between lower and upper tiers, specifically how a Tier-3 or Tier-4 level laboratory can supplement and support local Tier-1 and Tier-2 services respectively. Likewise, in addition to the proposed support infrastructure, ‘C’ also reveals how higher tiers can function together within a defined service precinct, to accommodate high service demands and provide infrastructure support in terms of service back-up and disaster recovery.

To gain understanding of the extent, distribution and efficiency of the existing NHLS CD4 laboratory service network, all NHLS Corporate Data Ware House (CDW) CD4 volumes and laboratory-to-result (LTR) Turnaround time (TAT) data (i.e. the time from first receipt of sample at registration onto the Laboratory Information Management System (LIMS) to time of authorization of results) was reviewed for the three-year period from April 2009 to April 2012. As it was assumed that there was no restriction of samples submitted for testing during the period of review, the historical volumes recorded were assumed to reflect clinics CD4 testing ‘needs’ (Table 1 reflects current proportional requirements). Existing laboratory CD4-testing capacities (and redundancy of service to accommodate possible increases of service needs) were determined by comparing the number and type of equipment placed in each laboratory to the respective volumes of CD4 test requests, per site (Table 2). Previously established GPS coordinates (latitude and longitude) of all referring NDOH health care facilities per district[19], as well as the location of existing NHLS laboratories (CD4 testing and non-CD4 testing facilities) were collated. ArcGIS (ESRI ( Johannesburg, South Africa) was used to generate maps of the GPS coordinates of all referring health facilities and/or existing NHLS general or CD4 laboratories (for Figs. 1-3 and Fig. 4b) and plot the relationship to different aspects of operational CDW CD4 data including average volumes of tests and TAT per district. Specifically, volumes of CD4 tests per testing site were linked to the geographical context of the referring CD4 work load i.e. the respective district from which the CD4 test requests came (as an indication of ‘demand’ for testing in any given area, Fig. 2). LTR-TAT were also analysed in the context of referring facility (insert Fig. 2) and averaged for each district. Data about total TAT, i.e. from sample draw to having a report in-hand, were not available and therefore could not be included. The extent of existing CD4 service coverage (at 2012) was estimated by creating a service radius (denoted a ‘service precinct’) around existing CD4 testing facilities. This enabled identifying the location of those health care facilities/clinics that lay outside of existing service precincts, without reasonable (100km) access to a local CD4 service. This exercise also facilitated identifying where placement of additional testing facilities/tiers would improve local TAT to <24 hours. The same exercise was also used to identify those over-serviced areas (e.g. Fig. 3, red circles) that could benefit from consolidation of services (e.g. to save costs of staffing, reagents, infrastructure).

**Table 1:** Relationship between CD4 tiers and NDOH Health Care Facilities.

		Applicable CD4 facility tier					
		Tier-1	Tier-2	Tier-3	Tier-4	Tier-5	Tier-6
		True POC	POC-Hub	Community Laboratories	District Labs	Centralized ‘Metro’ Labs	Reference/Super-Lab
*NDOH Health Care Facility type(20)	Proportion offering ART	<10^§^	<30-40^§^	≤150^§^	≤300^§^	>300^§^	>600^§^
Mobile Clinic	19%	Yes	Yes	Yes			
Satellite Clinic	4%	Yes	Yes	Yes	Yes		
Clinic (PHC), open 4 days per week	65%	Yes	Yes	Yes	Yes	Yes	
Community Day Centre (CDC), open 5-7 days per week	1%		Yes	Yes	Yes	Yes	
Community Health Centre/Hospital, open 24 hours, 7 days per week	5%			Yes	Yes	Yes	
District Hospital (Level 1)	5%			Yes	Yes	Yes	
Regional Hospital (Level 2)	1%					Yes	Yes
Tertiary Hospital (Level 3)	0%					Yes	Yes

Table showing the integration of the category of Health Care Facility (NDOH ‘Classification of Health Care Facility’ [20] offering ART) in relation to the proposed tier of CD4 service testing centre required to match and accommodate referred numbers of CD4 tests.

*testing facility framework and proportion offering ART; **^§^**sample testing capacity per day.

**Table 2:** Description of Proposed CD4 Testing Tiers.

	Tier-1	Tier-2	Tier-3	Tier-4	Tier-5	Tier-6	
	Proposed	Proposed	Proposed	Existing	Existing	Existing	
Tiered Facility detail	True POC	POC Hub	Community Labs	District Labs	Metro Labs	Reference Center	Grand Total
Average number of clinics serviced per testing facility	1	<10	11–50	51–100	>100 (max. noted here = 294)	>500 if super-lab	NA
Number of proposed sites	22	22	21	18	20	1	**104**
Daily volume capacity (8 hour day)	<10 (typically <5)	<30-40	<150	<300	>600	0	NA
Current maximum instrument capacity (8 hour day)	10	30	150	384	768	>1536 if super-lab	NA
Typical number of instruments	1*	1–2*	1–2*	2–4*	4 or more*	4 or more*	NA
Proposed % of samples tested per tier, per year	2%	3%	10%	15%	70%	NA	**100**
Projected number of samples per annum based on annual data 2012/13	78 000	117 000	390 000	585 000	2 730 000	NA	**3 900 000**
Instrument definition	POC/near patient device	POC/near patient device	Low-Medium§	Medium-High§	High§	High§	NA
Potential Commercial instrument supplier or equivalent	ALERE Pima or BD FACSPresto	BD FACSPresto, BD FACSCount or BC AquiosCL	BC AquiosCL and/or BC XL MCL	BC AquiosCL and/or XL MCL or MPL/CellMek	BC MPL/CellMek	BC MPL/CellMek	NA
Estimated Cost per Test in USD(18) based on BC pricing as at 2012	**$32.32**	**$15.88**	**$7.42**	**$6.24**	**$5.37**	NA	NA
Estimated annual national costs if **widespread POCT** is implemented to extend service coverage to existing laboratory service**^§§^**	**$18 907 200** (585 000 POC tests required to extend services; assuming no Tier-2 or Tier-3 services)	-	-	$3 650 400	$14 660 100	NA	**$37 217 700**
Estimated Annual National Cost to extend service coverage **applying full ITSDM** in USD (18)	$2 520 960	$1 857 960	$2 893 800	$3 650 400	$14 660 100	NA	**$25 583 220**
[ZAR**]	[R 27 730 560]	[R 20 437 560]	[R 31 831 800]	[R 40 154 400]	[R 161 327 100]		[R 281 415 420]**
							(saving $11 634 480 or R125 009 280)

Table outlining proposals for CD4 tiers, indicating number of sites, volumes (workload) per day and per annum, the number of clinics serviced, proposed platforms/instruments for testing and estimated costs.

§ Flow Cytometer systems with automated sample preparation systems accommodating testing volumes; *dependent upon organizational capacity planning and disaster recovery planning; **^§§^**Assuming widespread POC services are used to supplement existing laboratory services (i.e. no extended laboratory services at Tier-2 lab-supported POC-HUBS or Tier-3 community laboratories). **ZAR/USD exchange of R11/USD1 as at November 3^rd^, 2014. Abbreviations: BC, Beckman Coulter, USA. BD, BD Biosciences, USA. NA, not applicable.

Site-visits were undertaken by the CD4 team of the NHLS National Priority Programmes unit, together with local NHLS business- and laboratory-managerial staff, to obtain relevant service information about all CD4 laboratories and/or where applicable, confirm the locations of referring health care facilities and general pathology NHLS laboratories. This information enabled interpretation of analysed data and facilitated identifying possible new sites where implementation of local services could improve local TAT. Details included, information about existing NHLS laboratory infrastructure (i.e. buildings, air conditioning, benches etc.), capacity (staffing and existing CD4 equipment, if any), laboratory space (for expansion of services), local health care facility/clinic needs, as well as local geography, roads and supply services like water, electricity, amongst others. Specific service tiers were defined and weighted according to historical volumes of tests processed. Proposals for new sites of a specific tier were determined by the number of referring health-clinic facilities serviced in the area, historical (poor) TAT (exceeding the 24–48 hour target), whether there was a local CD4 laboratory service (or not) and proximity to the nearest CD4 or general NHLS services laboratory (which may have been in the same district or in an adjacent district). Published categories of national health care facility [20] (with special reference to facilities offering ART, see Table 1), recently recommended service levels [21] and established reliable laboratory quality systems [9], [22], [23] were also considered to the allocation of an appropriate tier of service to meet local CD4 testing demands.

## Results

### Review of general pathology services and CD4 workload across South Africa, within 9 provinces and 52 districts

The relative location of ∼4756 referring health care facilities (Fig. 1; black dots) in relation to 266 existing NHLS laboratories [24] (as green dots), reveals a relatively broad geographic distribution of general pathology service laboratories across South Africa. Services are focussed around the more densely populated areas with higher HIV prevalence, predominantly in the North-Eastern and Eastern pasts of the country, with an additional dense service focus in the South Western Cape. Historically-placed testing facilities in the latter regions have resulted in some over-servicing, with many testing facilities in close proximity to each other); in contrast, central areas of the country have considerably fewer health facilities and even less supporting laboratories.

During the period from 2009–2012, approximately 3.8 million PLG CD4 tests (Beckman Coulter, Miami, FL, US) were received at ∼60–70 designated NHLS CD4 facilities (some CD4 laboratories were shut down and others opened during the period of review, see Fig. 2, green dots). The majority of CD4 requests (∼67%), were referred from small primary health care facilities/clinics, with only 27% of all test requests originating from local on-site/hospital-based clinics, the latter also the location of most of the NHLS CD4 testing facilities (Fig. 1 insert). The remaining referred CD4 workload was referred from community clinics (8%), with the last 1% coming from parastatal institutions viz. prisons, military centers, amongst others.

Fig. 2 reveals the average daily work volumes (workload) of CD4 tests referred, across 52 districts and nine provinces in South Africa. The map is colour graded (as a ‘heat-map’) according to volume of work referred within a specific district; current geographic location of NHLS CD4 laboratories is shown in Fig. 2 (green dots, as at 2012). Despite the large proportion of referring primary health care clinics comprising the bulk of the annual work load, the highest demand for testing daily was typically noted in individual large community or hospital-based clinics within metropolitan areas. The volumes of CD4 tests thus varied considerably within any defined district depending on the proximity to a metropolitan centre or hospital. Whereas volumes could exceed 17 000 requests per year from an inner city clinic (e.g. in Gauteng, and within 10 km of three large referring hospitals), within the same district, outside of the main metropolis, as in some very remote areas, lower annual CD4 test-request volume averages of less than 5 per year were also seen. A separate review of the 20 busiest HIV/AIDS clinic sites referring the highest number of CD4 samples per day, revealed that eleven of these sites had immediate on-site access to a laboratory CD4 testing facility, with the remaining nine were in very close (5 km) radius of a testing facility.

### Review of laboratory turnaround times (TAT)

Fig. 2 (insert) reveals the percentage of reported results available with a 24–48 hour laboratory TAT, averaged over the 2009–2012 period, per district. This analysis reveals that in at least 75% of districts (39 of 52), between 96 and 100% of CD4 results were available for clinical use within a 24–48 hour TAT. A further 11% of districts received 86–95% of results within a 24–48 hr TAT. More remote and geographically challenging regions had CD4 laboratory TAT exceeding 48 hours; between 35 to 85% of samples were reported within a 48-hour TAT (but comprised just 8.2% of the national annual workload of 3.8 million tests, see Fig. 2 insert). Areas included Waterberg, Mopane, uMkhanyakude, Ruth-Segomotsi Mompati, Namakwa, Cacadu and West Coast districts. The furthermost northern district, Vhembe, (comprising 2.9% of the annual national work load) had the longest reported TAT; approximately only 34% of results were available within a <48-hour NHLS TAT (far Northern Limpopo, Vhembe district). Areas west and north-west of the country reflect lower volumes of requested CD4 tests (but also have lower population density and lower HIV prevalence (3)). Although there are general pathology services in these latter areas (Fig. 1), some districts lacked a local CD4 testing facility (see Fig. 2 where these districts are outlined in light blue) and had a smaller proportion of CD4 results available within the specified 24–48-hour LTR TAT (see Fig. 2 insert). CDW data and site visits confirmed that CD4 tests from these areas were referred to nearest testing facility in adjacent districts; often more than 100–200 km away and where longer LTR-TAT were recorded. For example, delays occur in the LTR-TAT in the Pixley ka Sema, JT Ghatsewe, Xhariep, and Lejweleputswa districts as the CD4 testing is typically referred (inland in this instance and outside of 100–200 km service precinct) into the Motheo, Siyanda and Francis Baard districts respectively (i.e. the Upington, the Pelonomi, Kimberly CD4 laboratories being respectively 433 km, 365 km and 243 km away).

### Review of existing laboratory services and capacity

In all CD4 laboratories, instruments and sample preparation equipment placed in sites met service needs, as determined by volumes of tests requested. Most laboratories processed more than 100 samples per day, with just under 20% of existing laboratories processing less than 100 samples per day (but usually more than 50). In the main metropolitan areas (including Gauteng, Western Cape, the Free State and KwaZulu-Natal), highly centralised ‘metro’ laboratories frequently processed in excess of 15 000 samples per month, with some metro-laboratories in Gauteng exceeding 25 000 samples per month. Comparison of workload volume to instrument capacity revealed capacity redundancy and reflected year-to-year NHLS operational planning for predicted NDOH service growth [6] and NHLS disaster recovery planning in the event of laboratory/instrument failure (downtime).

### Plotting service precincts of current testing laboratories

Fig. 3 reveals the estimated CD4 service coverage across South Africa during the study period. Relative Euclidian 100 km travelling-radius plotted around each established CD4 facility to establish respective ‘service precincts’ of each of the 60+ NHLS CD4 laboratories, revealed that most clinics across South Africa had reasonable access to service (Fig. 3). Over-subscribed areas with redundancy of capacity and/or staffing shortages, which could benefit from consolidation of service, were noted (Fig. 3, circled areas). More specifically, under-subscribed areas and ‘gaps in service’ were revealed, frequently in more remote areas (see Fig. 3 where ∼10% of clinic facilities fall outside of defined service precincts) and where implementation of additional Tier-1, Tier-2 or Tier-3 services (Fig. 4a), in closer proximity to health care facilities, could improve service delivery and TAT (also refer to Fig. 2 which reveals districts without local CD4 services). Clinics in these areas currently rely on the NHLS referral network; CD4 testing is performed in an adjacent district, many farther than 100 km away from their nearest respective testing laboratory. Extending travelling distances to a 200 km service precinct radius (precinct map not shown), still revealed many clinics (>50) that were further than 3–4 hours' drive to a testing facility.

### Integrating CD4 testing tiers ensure full service coverage

An integrated tiered service delivery model (ITSDM) incorporating six service tiers (details outlining characteristics of each tier can be seen in Table 2 with graphical representation in Fig. 4a) was required to ensure that CD4 testing is accessible at all South African health care facilities (Fig. 1) within a target TAT of <24–48 hours, irrespective of geographic location of the test request, and contain additional costs. A schematic of the ITSDM (Fig. 4a) and how the service tiers, including two POC service tiers, integrate with each other to ensure wide national access to CD4 services (Fig. 4b), the area within each tier representing the increasing number of health facilities (and hence volumes of referred tests) serviced. An additional single encompassing sixth ‘reference centre’ tier is recommended for coordination, harmonising and standardizing [21], [25], [26] of CD4 and related services (see Fig. 4a).

The first tier (as Tier-1) is mainly reserved for remote, “hard-to-reach” ART clinics/areas without reasonable access to a laboratory-based service, providing dedicated POC testing as a means of providing access to service, in a single ART providing site. Specific categories of POC tests and/or devices are defined for use in this context[21]; facilities will be able to perform up to 10 CD4 tests per day. Actual site-by-site analysis of historical CD4 test volumes in individual health care facilities suggests that test volumes will be <5 tests per day, only four days of the week, in keeping with services provided for in 65% of health facilities offering ART [20]. Attending nursing/clinic-support staff will most likely manage and conduct testing, as well as perform manufacturer directed quality control.

Tier-2 is an extended POCT tier; a small co-operative ‘POC-hub’, or mini-laboratory, servicing up to 8-10 referring clinics (within a 10-20km radius) where CD4 and/or tuberculosis testing can be implemented into an existing community laboratory that already provides baseline general pathology services, including basic haematology, chemistry (alanine transferase) and sexually transmitted disease (STD) testing required for ART initiation [17]. Using true CD4 POC technologies or operator-independent devices, historical volumes data suggests that the Tier-2 POC-hubs will require capacity to test between 5 and 40 CD4 samples per day, depending on service demands of the specific service precinct, and make use of existing NHLS laboratory transport or IT logistics to collect samples and distribute reports respectively (Fig. 4 reveals ∼22 proposed sites (yellow zones). Ongoing quality of testing of Tier-1 and Tier-2 will be monitored and managed through the closest higher tiered facilities, mostly likely local Tier-3 or Tier-4 laboratories (proposed location of ∼22 sites and examples of their relationship to higher supporting tiers can be seen in Fig. 4b).

This study revealed that many provincial districts did not have a local CD4 laboratory (lacking in 14 of 52 districts, Fig. 2, light blue outlined) with poorer local TAT (Fig. 2 insert). Historical volumes of requested tests suggest that the LTR-TAT of these districts can be improved extending CD4 services into Tier-3 facilities. Typical Tier-3 laboratories would utilize semi-automated testing systems (XL/MPL system already in use in the NHLS) [27] or smaller, fully automated/operator-independent flow analysis systems [27], [28] and process up to 150 samples per day to accommodate the volumes of tests expected from up to 100 health-clinics. Tier-3 services can be implemented into established small district and/or community NHLS laboratories that currently provide general pathology services (Fig. 1), with established building, logistical, information technology (IT) and staffing infrastructure.

Laboratory Tier-4 and Tier-5 meet the increasing service demands and higher workload volumes of the main metropolitan areas. Tier-4 laboratories are smaller sites, just outside of major metropolitan areas servicing up to 100 referring health clinics and performing up to 300 tests per day, using a combination of fully-automated, semi-automated testing systems or operator-independent flow cytometry based testing technologies, similar to that described for Tier-3 labs. Approximately, twenty-one Tier-4 testing laboratories are proposed here to meet the current needs of the South African service (Table 2).

Tier-5 services mainly covers dense populated areas, with highest service demand in and around large cities; accommodating 300–1500 tests per day and serving more than 200 health facilities. Fully-automated, walk-away systems for sample preparation and test analysis are used to meet the expected high required daily testing volumes of more than 350 tests (usually more than 650 based on historical data). One or more Tier-5 facilities may be required to meet the service demands in any given region (see red circles on Fig. 3 as examples). Approximately twenty Tier-5 laboratories are proposed for South Africa in this study, the majority already in existence (Fig. 2).

Tier-6 represents a national reference/‘monitoring and evaluation’ center responsible for coordination, harmonization and standardisation of testing, as well as coordination and organization of training and quality control across a national network of laboratories and related testing sites. Depending on local organization, Tier-4 and Tier-5 labs may potentially be consolidated into one large centralized laboratory (‘super-laboratory’/Tier-6 level), depending on efficiency of local transport systems and availability of technical staff to run these centers (an example of areas that would benefit from consolidation of services, see Fig. 3, circled areas). Support for all sites is envisaged from an integrated hierarchical support base of higher ‘parent’ tier sites supporting lower tiers of service, depending on the geographical location of the particular lower site and its nearest corresponding higher CD4 tier (see Figs. 4a and 4b).

### Impact on service delivery and cost

Fig. 4a reveals how increasing tiers of service provide for levels of increasing work load and from an increasing number of varying referring health-clinics (the hierarchical structure and function of clinic services is outlined in Table 1). Specific details about how the ITSDM can be applied in South Africa to ensure ‘full service coverage’, decentralising additional new proposed services across Tier-1, Tier-2 and Tier-3, is outlined in Table 2, (including the numbers of sites required within each tier to facilitate ‘full coverage’ with wider, more accessible CD4 services in South Africa). Detail is also provided in Table 2 of estimates of total number of tests that can be anticipated at each tier (existing Tier 4 and Tier-5 and proposed Tier-1, Tier-2 and Tier-3) and the respective tier calculated cost-per-test [18]. Currently in South Africa, the servicing of the historical 380 000 CD4 tests are tested in equivalent Tier-4 and Tier-5 laboratories, with a historical baseline cost of ∼ between $20 and $23 million. To expand services and enable national ‘full-coverage’, aiming at a universal 24–48 hour LTR-TAT, varying permutation of Tier 1, Tier-2 and Tier-3 services can be implemented, at varying additional cost. The model can thus be applied in different ways, by adding one or more of the proposed lower tiers (Tiers 1 to Tier-3) to the existing Tier-4/Tier-5 network; how the ITSDM is applied however, changes programmatic costs dramatically. For example, if the existing laboratory system (with Tier-4 and Tier-5 laboratories) is supplemented with only Tier-1/POC sites to extend services, then ∼585 000 POC tests at a cost of $32.32 per test is needed; an additional amount of ∼$18.9 million is therefore required to meet the stipulated LTR-TAT of <24 hours and ensure ‘full coverage’. However, distributing the additional CD4 services and spreading the load to small laboratories (mini-laboratories at Tier-2 and community laboratories Tier-3), and reserving the Tier-1/POC for ‘hard-to-reach’ sites without reasonable access to a laboratory (Tier-1), can markedly reduce total programmatic overheads of expansion. The 24-hour TAT requirements are met with this approach and the extension to CD4 services costs just ∼$7.2 million, potentially saving programmatic costs of ∼$11.7 million [18]. The implementation of the POC-hubs instead of widespread Tier-1 services costs $1.86 million and potentially reduces POC costs by ∼$1.86 million in the South African programme. The biggest saving is associated with introducing decentralized laboratory testing at Tier-3 level. Although 38% higher ($2.05 more) than the cost of performing a CD4 in a highly-centralised laboratory, performing a CD4 in a Tier-3 laboratory costs just 23% of the cost of providing a CD4 in a Tier-1/POC site or half the cost of providing the same test in a POC-hub.

## Discussion

The analysis of work load volumes in each district (Fig. 2) enabled an indirect objective measurement of the daily clinic CD4 testing demands in any specific district/area (assuming that there were no restrictions on number of samples submitted for testing). Volumes of tests followed district HIV prevalence, with highest numbers of tests being requested across the three provinces reported to have the highest HIV prevalence [1], viz. Gauteng, Kwazulu-Natal and South Eastern Coastal areas (see Fig. 2). Despite that the specific LTR-TAT definition used for this study was not ideal, in that data was not available concerning the total TAT from the point of venesection to the point of delivery of the report, review of the LTR-TAT data did enable important insights and conclusions to be drawn about trends of TAT and assessment of whether the state-driven-service met service demands (as evidenced by the historical test volumes), as well as the requirements of the NDOH treatment algorithm [29] (where patients are required to return for their CD4 results in 7 days). A largely reliable CD4 service was noted across the country. Eighty nine percent of districts, including the areas with highest test volumes, were able to fulfil the NDOH CMMT programmatic and treatment algorithm CD4 requirements. Most districts (and most hospital clinics) were therefore able to access patients CD4 results, certainly on the NHLS LIMS, with 1–2 days of receipt at the laboratory, within the NDOH algorithm time frame. Longer LTR-TAT, likely to impact on clinical management was however noted some parts of the country (Fig. 2 insert) highlighting local limitations. Specifically, vast distances between health care centers and current CD4 service testing facilities were documented in the centre of the country, where many referring health clinics lay outside of existing service precincts (see Fig. 3). Site inspections also revealed regional transport and logistical deficiencies, local (difficult) geography and poor or inconsistent supply of electricity services. Staffing and skills deficiencies, evident from site visits, were also noted to limit service and delay LTR TAT. Further, this study also revealed that at least six districts (noted in Fig. 2) with poor TAT, there was no local CD4 service laboratory (CD4 footprint).

It was evident from this work that at least six integrated hierarchical tiers of laboratory and/or POC services (the ITSDM) were required to deliver ‘full coverage’ with universal 24-48 hour LTR TAT, simultaneously providing access to CD4 services in high volume metropolitan areas, whilst at the same time, extending the service footprint of the laboratory network to areas where there was no reasonable access to a laboratory (by using POC technologies in hard-to-reach sites). By supplementing high volume testing laboratories with extended decentralised services at POC sites and in Tier-2 and 3 laboratories, a national laboratory service can potentially improve LTR-TAT (where TAT is not acceptable and exceeds HIV treatment algorithm requirements). In this way, ‘full–coverage’ of services is enabled, across a geographical wide area with vastly differing service needs.

Recent operational recommendations [21], define POC servicing in a single level. However, in a context of a country like South Africa where resources are limited and where POC testing has been shown to be up to four times the costs of decentralized laboratory-based testing, further taking into account possible disruption to existing clinical services where attending health care workers may be required to take on additional responsibility of near-patient pathology testing [30], [31], two levels of POC service are described in this ITSDM. An extended POC tier also utilises POC technologies but operates in a remote laboratory; this mini-laboratory/POC-hub services a cluster of health care facilities, all within reasonable 10–20 km travelling distance and POC tests are undertaken by a single operator. Consolidation POC of services into a single site assists with minimising logistics of blood collection [32], as well as limiting higher per test and capitation costs of implementation of CD4 testing [15], [18], [33] at the POC (see Table 2). This approach also takes cognizance of (lack of) available skills and limited laboratory and/or clinic infrastructure resources in poorly-serviced areas; testing need not necessarily be performed by qualified technologists, but may be done by suitably qualified dedicated technicians who would perform all testing using POC devices, consolidating on staffing costs. The quality control of capillary blood typically required for use in POC CD4 technologies, needs special attention [34], [35] and further research is necessary to develop systems that quality control accuracy and precision of capillary blood sampling as well as proper external quality assessment programs. Newer POC CD4 technologies [36], [37] do promise better controlled capillary sampling but field studies are outstanding.

Overall, the strength and sustainability of the ITSDM described, lies in providing a hierarchy of ‘parental’ support described above, to facilitate quality management across all tiers, but especially support the new Tier-1- 3 sites (including aspects such as daily quality control, staff training, and logistics of multiple finger prick and sample collection at POC sites). Quality management of the ITSDM can also be ensured through direction and supervision of services by a multi-skilled team of pathologists, scientists and technologists as the main custodians and supporting group for all CD4 services, including services at the POC. This is especially important where, for example, health care providers at the POC may be solely responsible for testing with no access to other support structures and would rely on local established laboratory infrastructure and technical expertise for testing and equipment maintenance, quality control as well as training and support. The ITSDM also provides for a harmonising and standardising, ‘monitoring and evaluation’ sixth tier. In the context of ideal and fully efficient transport and information technology (IT) logistics present in modern, first world centers, this Tier-6 could also represent a ‘super-laboratory’ where all testing is facilitated through a few strategically placed, very large ‘factory-like’ testing facilities.

A comparative summary-costing analysis focused on a single National Health Insurance pilot district (Pixley-ka-Seme district in the Northern Cape) [18], outlines details of respective costs of the POC Ters-1 and Tier-2, as well as related laboratory-based tiers, including capital (start-up) and on-going recurring costs. Specifically, this study reveals that higher Tier-1 true POC costs can be offset against savings made by consolidating some POC testing into collective ‘hubs’. An additional factor comes into play when procuring national services. In South Africa, economy of scale and fixed tender cost-to-company, per laboratory CD4 test, irrespective of the tier of laboratory employing the test, makes implementation of Tier-3 laboratories a very efficient option to save costs (see Table 2); the cost of providing a CD4 test in a community laboratory obviates the need to incur higher costs of POC services and is less than a quarter of the cost of providing a POC CD4. Tier-3 services can be also implemented making use of existing staff in sites (redundancy); training requirements are minimized, especially if operator-independent, ‘walk-away’ technologies are implemented [38].

Decentralised servicing using existing laboratory infrastructure within these districts also emerged as an important aspect of enabling service delivery and meaningful clinical TAT in undersubscribed areas. Certainly, in South Africa, where such infrastructure largely exists (as small rural general pathology laboratories), extending ART-based laboratory POC services or creating Tier-3 services in these existing laboratories that already offer general pathology services and have basic infrastructure, can provide a means to rapidly extend and consolidate accessibility to service. At the time of this analysis, an additional five new Tier-3 NHLS CD4 sites had been implemented (2014) with significantly improved LTR TAT to <than 24 hours [39], leaving 9 of 52 districts with limited or no access to a local CD4 service. The positive impact of extending laboratory coverage has been documented [39]; setting up a new Tier-3/decentralised laboratory at De Aar in Pixley-ka-Sema (a small town in a district that lies in a remote central part of South Africa with no prior CD4 laboratory), has enabled significant reduction in overall LTR TAT. In this district, pre-analytical TAT has been consistently reduced from>15–48 hours to <5 hours and overall LTR TAT to <18 hours, largely due to shorter sample travel times to the new local service laboratory in the district.

Finally, it is important to reiterate that the ITSDM is a pathology service delivery framework, utilizing a public health approach and proposes efficient use of national funding, to ensure that all patients across a national programme have reasonable access to a CD4 count service and that the results are available within the timeframe required by national treatment policy and algorithm guidelines [29]. It is thus not only a model for CD4 testing but for other HIV pathology services. The typically described role of providing POC CD4 in remote [12], [40] or in urban centers [30], [41], to improve and encourage enrolment of patients onto ART, is not a role of POC services in the ITSDM. It is therefore important to acknowledge that although the model incorporates use of POC CD4 testing to extend service delivery, it does not provide for universal immediate POC access to CD4 reporting. In the ITSDM, laboratory-based testing is extended into smaller existing laboratories using walk-away operator-independent laboratory systems, that require less technical skills and enable a CD4 service with a 24–48 hour TAT to the referring clinics. This is a considerably cheaper way to extend services than implementing widespread POC testing i.e. putting POC technologies into all sites currently within range of a community laboratory service. Table 2 reflects that implementation of the ITSDM with Tier-3 services, as opposed to extending services with widespread implementation of Tier-1 POC testing, can potentially save South Africa R125 million on CD4 servicing costs. However, if required, and where NDOH budgetary funding permits, immediate POC CD4 servicing to encourage improved enrolment onto ART programmes can be built into the ITSDM. Ultimately, understanding programmatic costs (see Table 2) and plans, the availability of programmatic funding at provincial or district level, and making best use of existing resources (especially the existence and location of current small routine pathology laboratories in any given region in South Africa), will determine the combination and permutations of the different tier facilities that are ultimately implemented to ensure CD4 ‘full service coverage’.

## Summary and Conclusion

A demand based, six-tiered hierarchical ‘full service coverage’ approach is described that accommodates delivery of services, where wide geographic distances between services and health-care facilities exist with differing service workload requirements, in a single service delivery model. Centers with significantly higher service demands exceeding 15 000 samples per month, in metropolitan areas, are catered for whilst in remote sites, centres without reasonable access to a laboratory with low-volume testing requirements of <5 tests per day, are accommodated with POC service. Six tiers, with specific testing volume thresholds, are defined in the service framework. These include decentralized POC (Tier-1), Point-of-care (POC) Hubs (Tier-2) and community laboratories (Tier-3), with centralized testing accommodating higher numbers of referring clinics and higher workloads at district (Tier-4) and ‘metro’ (Tier-5) laboratories in metropolitan areas. The important role of additional POC tiers (as Tier-1 and 2) and decentralised laboratory service tiers (Tier-3) is highlighted, providing for services in deficient ‘hard to reach’ sites areas and making use of existing infrastructure as a valuable resource. The value of a supernumerary coordinating-umbrella sixth tier, responsible for coordinating, harmonization and standardisation of testing, training and quality control across the national network of laboratories and related testing sites, is noted. Understanding the unique role of each service tier and the value of integrated ‘parent-tier’ support, the related economic benefits of fixed-cost laboratory-based testing with cross-subsidization of higher costs across to provide POC service tiers where needed, can ultimately ensure an equitable, accessible, sustainable and scalable CD4 service that is both affordable and saves programmatic costs.
